# Supplementary material for: MultiMatch: geometry-informed colocalization in multi-color super-resolution microscopy
Source: Commun Biol. 2024 Sep 13;7:1139. doi: 10.1038/s42003-024-06772-8 (PMC11399439; doi:10.1038/s42003-024-06772-8)
Supplement: Supplementary file 1 — Supplementary Information [file 42003_2024_6772_MOESM1_ESM.pdf]

MultiMatch: Geometry-Informed Colocalization in Multi-Color Super-Resolution Microscopy

Julia Naas[1,2], Giacomo Nies[3,4], Housen Li[3,4], Stefan Stoldt[4,5,6], Bernhard Schmitzer[7], Stefan Jakobs[4,5,6,8] and Axel Munk[3,4*]

[1]Center for Integrative Bioinformatics Vienna (CIBIV), Max Perutz Labs, University of Vienna and Medical University of Vienna, Vienna BioCenter, Vienna, Austria.

[2]Vienna Biocenter PhD Program, a Doctoral School of the University of Vienna and Medical University of Vienna, Vienna, Austria.

[3]Institute for Mathematical Stochastics, University of Göttingen, Göttingen, Germany.

[4]Cluster of Excellence "Multiscale Bioimaging: from Molecular Machines to Networks of Excitable Cells" (MBExC), University of Göttingen, Göttingen, Germany.

[5]Department of NanoBiophotonics Max Planck Institute for Multidisciplinary Sciences, Göttingen, Germany.

[6]Clinic of Neurology, University Medical Center Göttingen, Göttingen, Germany.

[7]Institute for Computer Science, University of Göttingen, Göttingen, Germany.

[8]Fraunhofer Institute for Translational Medicine and Pharmacology ITMP, Translational Neuroinflammation and Automated Microscopy TNM, Göttingen, Germany.

*Corresponding author(s). E-mail(s): munk@math.uni-goettingen.de;

Contributing authors: julia.naas@meduniwien.ac.at; thomas.nies@uni-goettingen.de; housen.li@mathematik.uni-goettingen.de; stefan.stoldt@mpinat.mpg.de; schmitzer@cs.uni-goettingen.de; sjakobs@gwdg.de;

Supplementary Note 1

Network flow implementation scheme

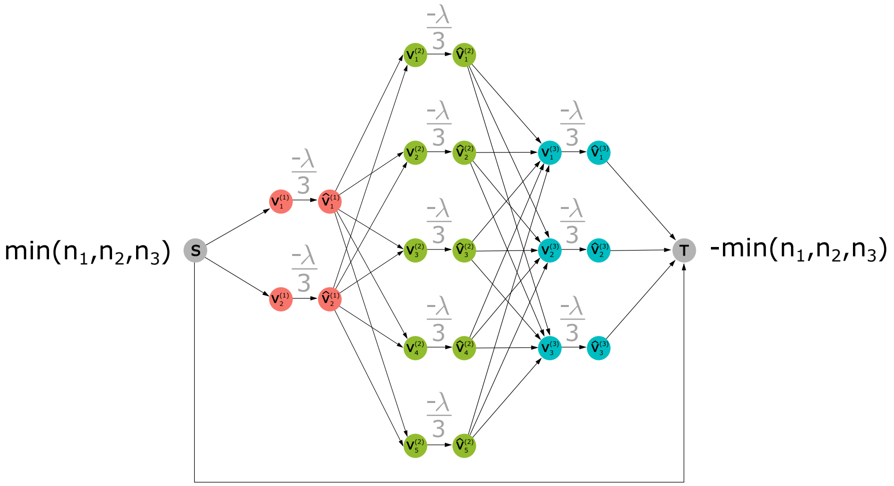

Supplementary Figure 1: Scheme of the implemented min cost network flow problem in MultiMatch as described in section 'Network Flow Formulation' (Methods), for the detection of chain-like particles for three color channels, where n_i is the number of particles detected in channel i.

Supplementary Note 2

Output of MultiMatch Mode II on the experimental STED images

To compare MultiMatch Mode I and II, we repeated the colocalization analysis of experimental STED image settings with MultiMatch Mode II (see Supplementary Figure 2). As expected, the mean relative abundance curves $\hat{\mathbf{w}}$ and the corresponding estimation results of $\hat{\mathbf{n}}$ are comparable to the results reported by Mode I (see Figure 4 for analysis output by Mode I). However, since Mode II does not prioritize the detection of triplets, unlike Mode I, the triplets frequencies are more underestimated in Setting 3, where only triplets

should occur in the image. The maximal relative mean abundance of detected ABC triplets, which is attained for colocalization threshold $t = 10$ pixels is $w_{ABD} = 0.77$. For MultiMatch Mode I it is closer to the (by experimental design known) truth of having triplets only, by reaching a maximal relative abundance of $w_{ABC} = 0.8$ (see Figure 4).

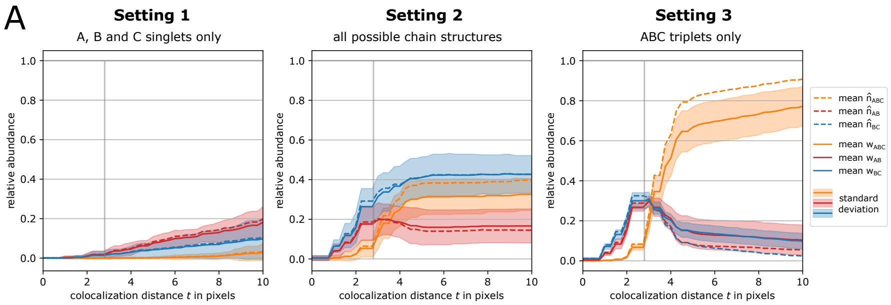

Supplementary Figure 2: **MultiMatch Mode II relative abundance curves $\boldsymbol{w}(t)$ for experimental STED images.** As Figure 4 but analysed with MultiMatch Mode II instead of Mode I: For each setting the solid curves are mean relative abundances with standard deviation bands across a range of colocalization thresholds t from 0 to 10 pixels (25 nm = 1 pixel). The abundances are scaled by the total number of points detected in channel B. Additionally, incomplete labeling efficiency (90% in each channel) corrected abundances are plotted as dotted curves. The true colocalization threshold of 70 nm within nanoruler structures is depicted as a vertical line. **A.** *Setting 1* contains singlet (22 independent experimental STED images), *Setting 2* triplet, pair and singlet (22 independent experimental STED images) and *Setting 3* triplet nanorulers only (12 independent experimental STED images).

Supplementary Note 3

Comparison of methods across colocalization thresholds

We also tested the performances of considered colocalization methods across different colocalization thresholds t, see Supplementary Figure 3.

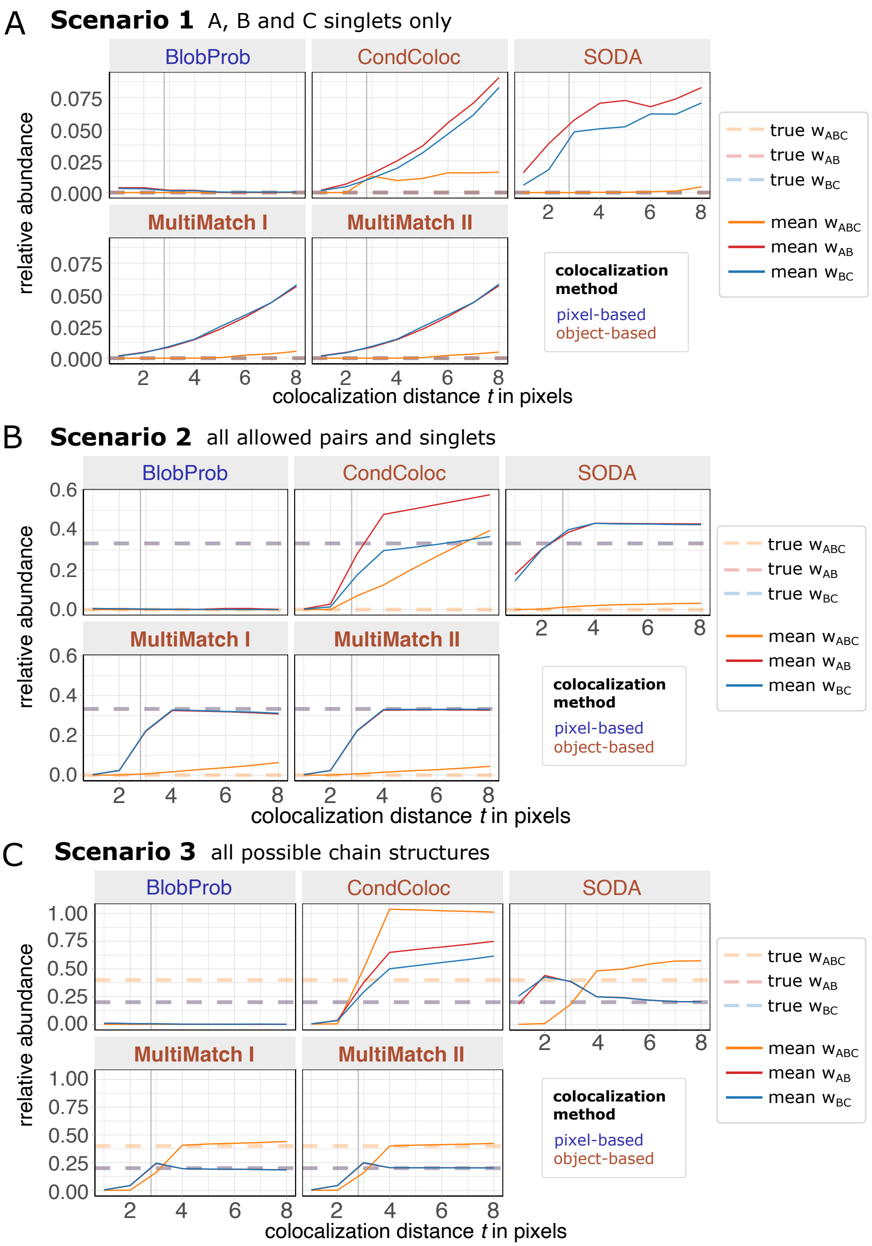
A
Scenario 1 A, B and C singlets only
BlobProb
CondColoc
SODA
MultiMatch I
MultiMatch II
rrelative abundance
0.075
0.050
0.025
0.000
2
4
6
8
colocalization distance t in pixels
true w_{ABC}
true w_{AB}
true w_{BC}
mean w_{ABC}
mean w_{AB}
mean w_{BC}
colocalization method
pixel-based
object-based
B
Scenario 2 all allowed pairs and singlets
0.6
0.4
0.2
0.0
C
Scenario 3 all possible chain structures
1.00
0.75
0.50
0.25
0.00

Supplementary Figure 3: **Simulation study for three-color microscopy images with three combinations of chain structures along different colocalization thresholds** t**.** In each Scenario 100 independent STED images and different abundances of triplets, pairs and singlets were simulated with 100% labeling efficiency (Methods). Mean relative abundances curves (scaled by the total number of points in channel B) are shown per colocalization method and chain structure. True simulated relative abundances are plotted as transparent, horizontal dashed lines. **A.** In *Scenario 1* only A,B and C singlets, in **B.** *Scenario 2* all possible singlets as well as AB and BC pairs and in **C.** *Scenario 3* ABC triplets, AB, BC pairs and A, B and C singlets were simulated.

Methods were evaluated on the colocalization grid $t \in \{1, 2, 3, 4, 5, 6, 7, 8\}$ pixels. We experienced, that BlobProb and ConditionalColoc were not directly applicable to a batch of images at once. In particular, BlobProb requires the user to load every image separately into an ImageJ/Fiji Graphical User Interface, where parameters as the colocalization threshold have to be adjusted by hand, respectively. For the MATLAB implementation of ConditionalColoc, all images within a simulation scenario had to be combined into a 'movieList', which could then be input as a whole for colocalization analysis. However, the analysis had to be performed separately for each colocalization threshold. With the runtime of 2 minutes per image and colocalization radius, as reported in the ConditionalColoc manual (https://github.com/kjaqaman/conditionalColoc), the evaluation of our simulation study with ConditionalColoc took about 1000 times longer than with our MultiMatch implementations (0.1 seconds per image and colocalization radius, see Methods Section 4.2).

Comparison with Nearest Neighbor Matching

In addition to the considered colocalization methods provided as packages, plugins or executable scripts, one can also consider greedy Nearest Neighbor Matchings as a compatible algorithm to MultiMatch. Nearest Neighbor Matchings can be implemented in several ways depending on the order of points within a channel and the order of channels being matched in pairs. For the method comparison below, we used the following implementation:

1. For each point in channel A, assign it to its nearest neighbor in B as soon as their pairwise distance is smaller than the colocalization threshold t. If matched, do not consider the respective B point for further nearest neighbor searches of channel A points. The match is stored as **AB pair**.
2. Repeat 1. to match points in channel B to their nearest neighbors in channel C. Respective matches are stored as **BC pairs**.
3. If an AB and a BC pair share the same B point, they are re-annotated into one **ABC triplet**.

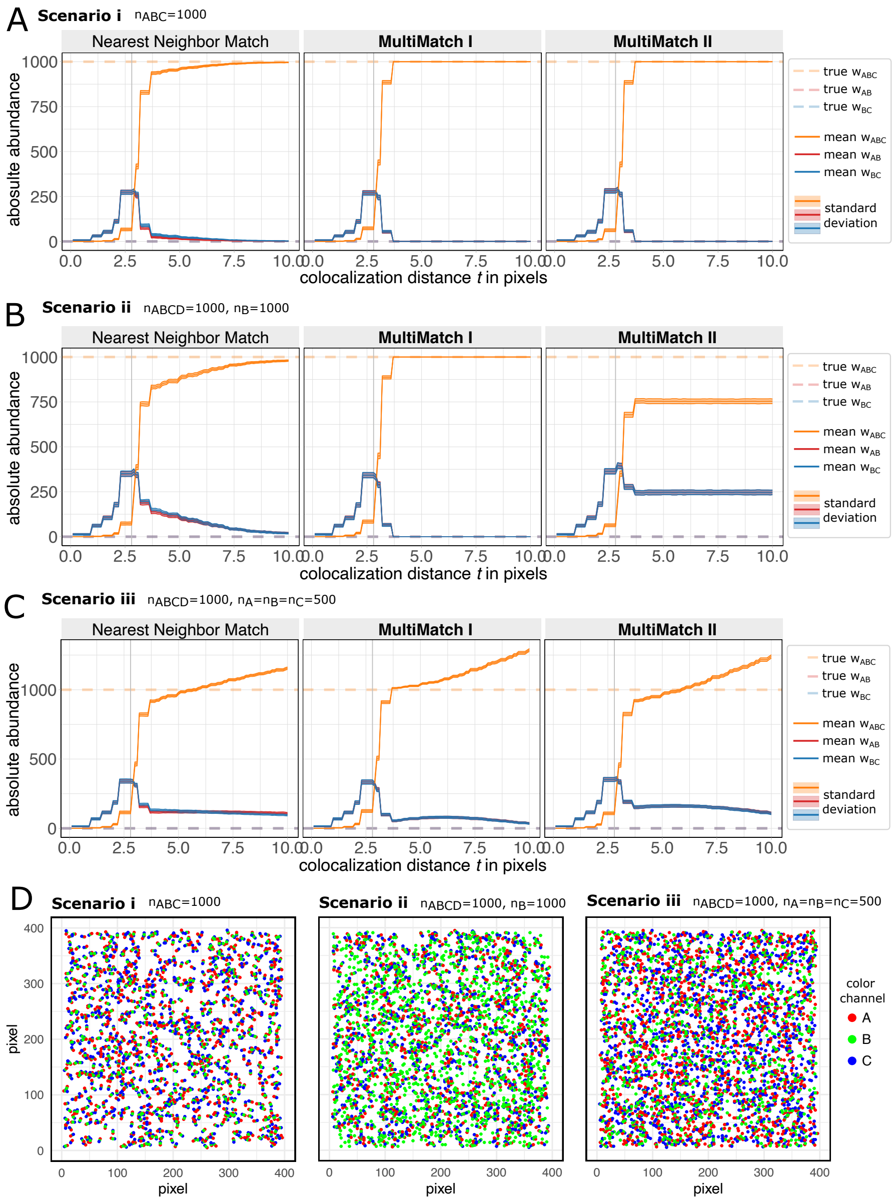

Supplementary Figure 4: Simulation study for three combinations of chain structures along different colocalization thresholds t. Mean absolute abundances curves with standard deviation bands are shown per method and chain structure. True simulated relative abundances are plotted as transparent, horizontal dashed lines. For each scenario 100 independent images were simulated. **A.** In *Scenario i* only ABC triplets and in **B.** *Scenario ii* ABC triplets as well as B singlets were simulated. **C.** *Scenario ii* ABC triplets as well as A,B and C singlets were simulated. **D.** Representative particle clouds for Scenarios i-iii.

We applied MultiMatch Mode I and II and the above described Nearest Neighbor Matching approach directly to the simulated point clouds without additional conversion to microscopy intensity images. To illustrate the differences between MultiMatch's global optimization procedure and the effect of local, greedy Nearest Neighbor searches, we chose to simulate settings with a high particle density and an especially high number of triplets:

Scenario i: 1000 ABC triplets only.
Scenario ii: 1000 ABC triplets and 1000 B singlets.
Scenario iii: 1000 ABC triplets, 500 A, B and C singlets, respectively.

As in all other simulation scenarios, the pixel size was simulated as 1 pixel = 25 nm, the image size was set to 400×400 pixels and the true colocalization distance was fixed to t = 70 nm. For each setting 100 images were simulated, but we directly evaluate the coordinates of simulated point clouds without further translation into an intensity image nor simulation of microscopy noise or point spread function convolution (see examples in Supplementary Figure 4D).

As can be seen in Supplementary Figure 4A, in Scenario i the Nearest Neighbor Matching approach underestimates the number of ABC triplets and overestimates the abundance of AB and BC pairs, although only triplets were simulated. For the a colocalization threshold of 4 pixels = 100 nm, the mean abundance detected by the Nearest Neighbor Matchings only reaches around 939.43 of 1000 simulated ABC triplets with a standard deviation of ca 0.197, while in both MultiMatch Modes I and II all 1000 simulated ABC triplets are recovered for every simulated image.

In Scenario ii we can showcase a similar behavior: Nearest Neighbor Matchings only reach a maximal average abundance of 838.71 out of the 1000 simulated ABC triplets for t = 4 pixels.

Additionally, we can observe that this setting is also challenging MultiMatch Mode II: Similar to Nearest Neighbor Matchings, MultiMatch Mode II underestimates triplets due to the disproportionate abundance of Type B particles and the dense particle distribution. Mode II finds more AB pairs with lower overall pairwise particle distances and therefore on average misses around 15% of all simulated ABC triplets in maximal colocalization threshold.

Interestingly, the shape of abundance curves for the Nearest Neighbor Matching is very similar between Scenarios i, ii and iii altough in the first we only simulated triplets and in the latter two we added different types and ratios of singlets. The Nearest Neighbor Matching approach does not only underestimate triplet abundances but also shows no clear plateau to discriminate between different colocalization structures. MultiMatch Mode I abundance curves, on the other hand, stabilizes at the correct abundance for triplet abundances for Scenario i and ii. If a random distribution of more than one type of singlets is present in the image (Scenario iii), singlets are matched as soon as the colocalization threshold is high enough. Still, one can observe that the abundance curve slopes visibly drop for $t \geq 4$ pixels.

Additional Four-Color STED Simulation Scenarios

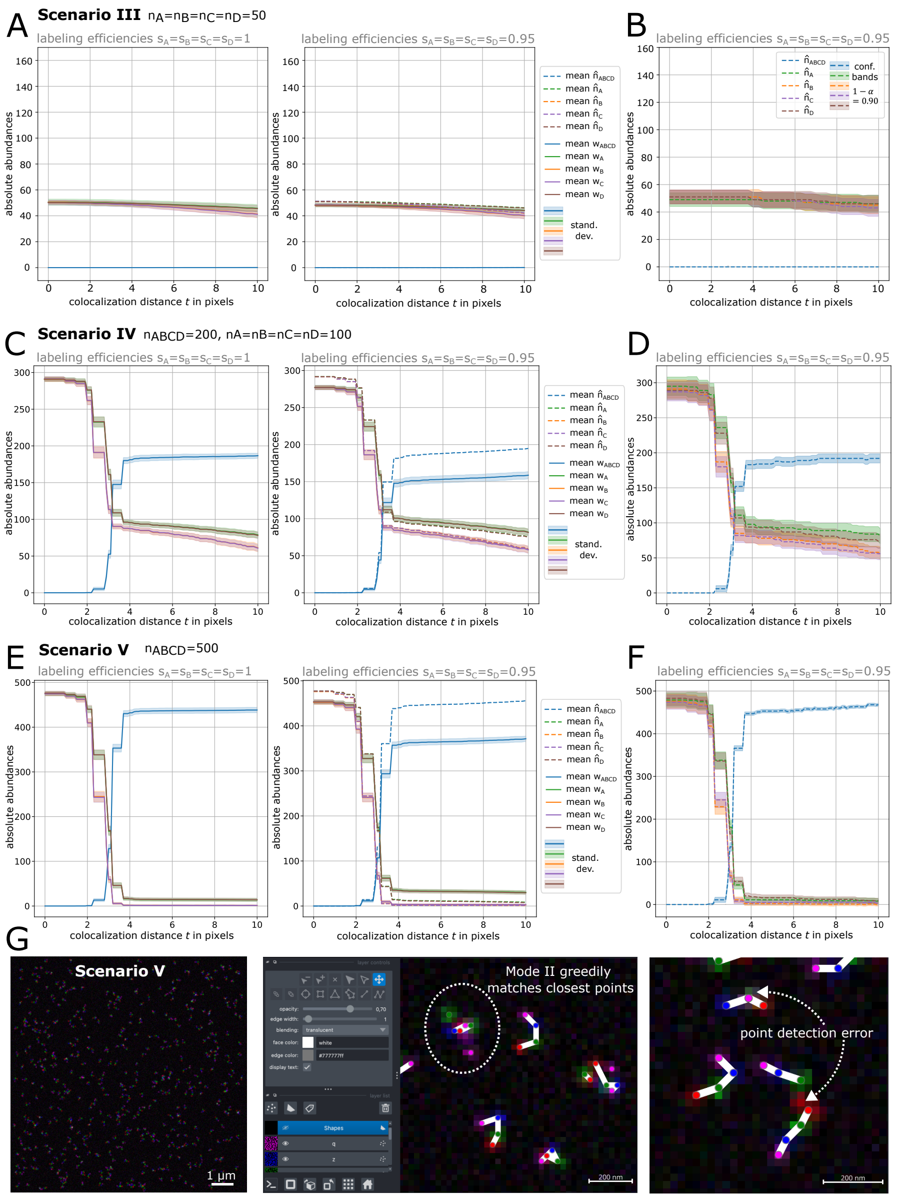

Supplementary Figure 5: **MultiMatch Mode II abundance curves $\boldsymbol{w}(t)$ and estimation results $\hat{\boldsymbol{n}}(t)$ for simulated four-colour STED images.** For each simulated scenario 100 independent images were simulated with complete labeling efficiency ($s_A = s_B = s_C = s_D = 1$) and with incomplete labeling efficiency ($s_A = s_B = s_C = s_D = 0.95$), respectively. Solid curves are mean absolute detected abundances with standard deviation bands across a range of colocalization thresholds t from 0 to 10 pixels (25 nm = 1pixel). Corrected abundances are plotted as dotted curves. **A.** *Scenario III:* Only singlets were simulated. **B.** For one exemplary STED image of Scenario III simulated with incomplete labeling efficiency, corrected abundance curves and corresponding confidence bands are shown. **C.** and **D.** show the same analysis as shown in A and B but for *Scenario IV:* ABCD quadruplets and A,B,C and D singlets were simulated. **E.** and **F.** show the same analysis as shown in A and B but for *Scenario V:* ABCD quadruplets only were simulated. **G.** Representative STED image for Scenario V with image details in the interactive napari viewer allowing a visual check of the image and MultiMatch output quality. For visualization purposes, contrast stretching and increasement of image brightness was applied.

Supporting Section 2.5, we additionally simulated the following three four-color STED image scenarios:

Scenario III: 50 A, B, C and D singlets, respectively, and no further quadruples, triplets nor pairs.
Scenario IV: 200 ABCD quadruples and 100 A,B, C and D singlets, respectively.
Scenario IV: 500 ABCD quadruples only.

From the respective MultiMatch Mode II results of those two simulations scenarios, shown in Supplementary Figure 5, one draws that abundance curves again are stabilizing after a colocalization threshold of $t = 4$ pixels. In case the detection of chain structures, here ABCD quadruples, is aggravated by incomplete labeling efficiency, our estimation framework leads to a consistent improvement of detection results towards the simulated ground truth.

For simulation Scenarios IV and V it becomes obvious, that due to high number of particles in the image, the simulated noise and according point detection errors, MultiMatch can not recover all simulated quadruples. In this case, the interactive napari viewer[1] can help to evaluate the noise level, point detection performance and matching results. For uniform noise levels and point detection errors across all channels, we recommend to evaluate channel-wise scaled instead of absolute abundance curves.

Supplementary Note 4

Simulation study of incomplete labeling efficiencies

We simulated incomplete labeling efficiencies by following the statistical framework developed in the Proof of Theorem 2, Part 1 in section 'Estimating the true chain-like particle abundances' (Methods): The numbers of detectable triplets, pairs and singlets $\boldsymbol{W}$ were simulated from true abundances $\boldsymbol{n}$ by drawing 10,000 values from respective multinomial distributions based on predefined staining efficiencies s_A, s_B, s_C (see multinomial model in Equations 12 and 13).All combinations of abundances and staining efficiencies that were simulated are listed in Supplementary Table 1, where we also recorded the respective empirical coverage of constructed joint confidence ellipsoids at a theoretical coverage of $1 - \alpha = 0.90$.

For $s_A = s_B = s_C = 0.95$ and $n_{ABC} = 500, n_A = n_B = s_C = 50$, matrix $\Theta\Sigma(\hat{\boldsymbol{n}})\Theta^T$ was invertable in every simulation and, as we can see in Supplementary Figure 6, simulated Ξ values are approximately chi-square distributed with 6 degrees of freedom.

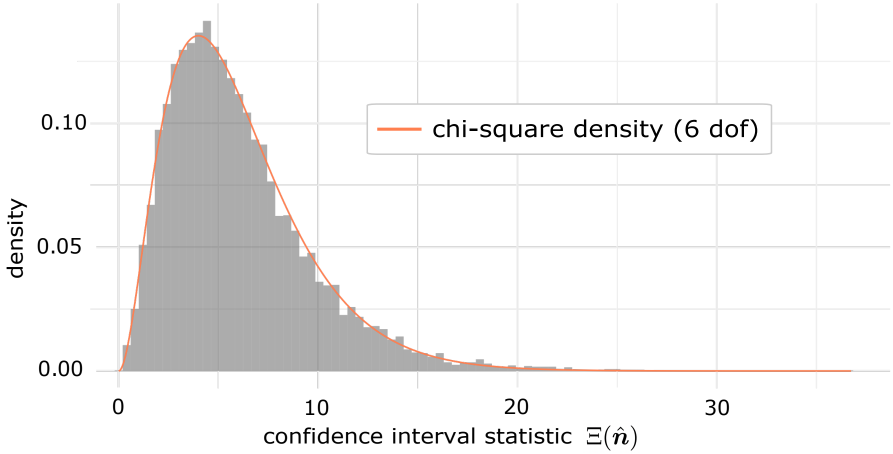

Supplementary Figure 6: 10,000 simulated independent Ξ values (see Proof of Theorem 2, Part 1 in section 'Estimating the true chain-like particle abundances' (Methods)) for simulation setting $s_A = s_B = s_C = 0.95$ and $n_{ABC} = 500, n_A = n_B = s_C = 50$ approximately follow a chi-square distribution with 6 degrees of freedom.

$s_A = s_B = s_C$	n_{ABC}	$n_{AB} = n_{BC} = n_A = n_B = n_C$	Empirical Coverage
0.80	50	50	0.8879
0.80	100	50	0.8884
0.80	500	50	0.8955
0.85	50	50	0.8893
0.85	100	50	0.8919
0.85	500	50	0.8897
0.90	50	50	0.8906
0.90	100	50	0.8984
0.90	500	50	0.8963
0.95	50	50	0.8898
0.95	100	50	0.8953
0.95	500	50	0.8940

Supplementary Table 1: Empirical coverage for the simulation of different triplet, pair and singlet abundances $\boldsymbol{n}$ and labeling efficiencies s_A, s_B, s_C at a theoretical coverage of $1 - \alpha = 0.90$.

Supplementary Note 5

Comments on the output from our usage of ConditionalColoc

We experienced that ConditionalColoc, although aiming to output probabilities, in some cases yields values greater than one and hence the errors in relative abundance detection are not bounded by one as well. In the following Supplementary Figure 7 we show the ConditionalColoc outliers not depicted in Figure 2.

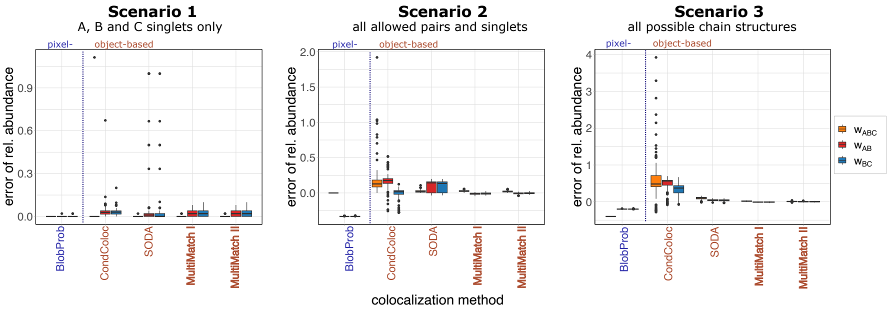

Supplementary Figure 7: **Simulation study for three-color microscopy images with three combinations of chain structures including ConditionalColoc outliers.** As Figure 2 but including outliers of ConditionalColoc resulting in errors in relative abundances greater than one. In each Scenario 100 independent STED images and different abundances of triplets, pairs and singlets were simulated with 100% labeling efficiency. **A.** Method specific boxplots of the errors in detected relative (scaled by the total number of points in channel B) structure abundances are displayed. The error is computed by subtracting true relative abundance from detected relative abundances. In *Scenario 1* only A,B and C singlets, in *Scenario 2* all possible singlets as well as AB and BC pairs and in *Scenario 3* ABC triplets, AB, BC pairs and A, B and C singlets were simulated.

References

[1] napari contributers. Napari: A multi-dimensional image viewer for python (2019). URL https://doi.org/10.5281/zenodo.8115575.
